# The pilot project of the National Cancer Network in Poland: Assessment of the functioning of the National Cancer Network and results from quality indicators for lung cancer (2019–2021)

**DOI:** 10.1186/s12885-022-10020-9

**Published:** 2022-08-31

**Authors:** Łukasz Trembecki, Aleksandra Sztuder, Izabella Dębicka, Rafał Matkowski, Adam Maciejczyk

**Affiliations:** 1 Department of Oncology, Wroclaw Medical University, pl. L. Hirszfelda 12, 53-413 Wroclaw, Poland; 2 Department of Radiotherapy, Lower Silesian Oncology, Pulmonology and Haematology Center, Pl. L, Hirszfelda 12, Wroclaw, 53-413 Poland; 3 Department of Chemotherapy, Lower Silesian Oncology, Pulmonology and Haematology Center, pl. L. Hirszfelda 12, Wroclaw, 53-413 Poland; 4 Breast Unit, Lower Silesian, Oncology, Pulmonology and Haematology Center,, Pl. L. Hirszfelda 12, Wroclaw, 53-413 Poland

**Keywords:** Lung cancer, Poland, National Cancer Network

## Abstract

**Background:**

This analysis presents the outcomes of the operations of the National Cancer Network (NCN) pilot project in Lower Silesian Voivodeship, Poland, for lung cancer for the period of 2019–2021. The results concerning measures of the quality of medical processes were analysed.

**Methods:**

Twenty-one measures used to gauge the quality of oncological care for lung cancer were assessed. Data collection and processing for the purpose of calculating the measures were carried out as part of the NCN pilot project based on the Regulation of the Ministry of Health enacted on 13 December 2018. The measures were calculated at the Voivodeship Coordination Center, and the data were derived from the centres included in the network in the area of the analysed voivodeship.

**Results:**

A total of 3,638 patients diagnosed with lung cancer were enrolled in the NCN pilot program during the analysed period. For 3 measures, out of 21, target values were obtained. For 2 measures, the values differed significantly from the assumed target value.

**Conclusion:**

In our opinion, the NCN pilot study, as a test of the network’s functioning, meets the assumed goal. The NCN assessment is based on, inter alia, analysis of the outcomes of oncological quality of care measures for lung cancer, and facilitates monitoring of the quality of medical services provided and the identification of areas for improvement. In addition, the pilot program, which will last until the end of 2022, will allow for further in-depth analysis regarding the network’s limitations before implementing the system on a national scale in Poland. This will be the subject of further investigation.

**Supplementary Information:**

The online version contains supplementary material available at 10.1186/s12885-022-10020-9.

## Introduction

Cancer has become one of the greatest challenges faced by medicine in the twenty-first century. Increased average life expectancy and exposure to carcinogens are the main factors related to the observed rise in the number of malignancies. Among them, lung cancer constitutes a significant problem for the Polish health care system. Between 2015 and 2019, lung cancer accounted for 17% and 9% of all cases of malignancy recorded in Poland in men and women, respectively. Currently – it is first cause of cancer deaths, both among men and women in Poland and one of the most frequent cancers [1]. To quote data concerning the projected epidemiology of lung cancer in Poland, by 2030, the number of new cases is expected to increase by 17% in men and 10% in women [2].

To improve treatment outcomes, in addition to high-quality medical services in the field of oncology, efficient coordination of diagnostic and therapeutic processes is necessary.

In early 2019, a pilot project for the National Cancer Network (NCN) was launched in Poland, with the goal of (among others) improving the coordination of treatment and introducing a system to monitor the quality of oncological procedures for the most common types of cancer [3].

The pilot project covers four provinces: Lower Silesia Province, Holy Cross Province, Pomerania Province, and Podlasie Province, with a total population of 7.6 million [4]. The aim of this paper is to present the initial results of the NCN’s functioning in the scope of lung cancer diagnosis and treatemnt for Lower Silesia Province between 2019 and 2021.

## Materials and methods

The pilot project for the Polish NCN is based on the Regulation of the Ministry of Health enacted on 13 December 2018; its goal is to assess the organisation and quality of oncological care within an oncology network operating in the provinces covered by the pilot project [3]. Patients diagnosed with lung cancer, breast cancer, colorectal cancer, prostate cancer, and ovarian cancer were included. In Lower Silesia Province, whose population is 2.9 million, data concerning lung cancer have been reported since 1 February 2019. The pilot project was originally set to end on 31 December 2021 [5]. However, under the Regulation of the Ministry of Health enacted on 23 December 2021, the pilot project has been extended until 31 December 2022 [6].

The network comprises a Regional Coordination Centre as well as first- and second-level support centres, which cooperate in the scope of oncological care provided to patients covered by the pilot project.

In Lower Silesia Province (the subject of this analysis), the Regional Coordination Centre’s role was entrusted to the Wroclaw Comprehensive Cancer Centre in Wroclaw as the centre with the highest capacity in terms of medical personnel and the ability to provide comprehensive oncological treatment (i.e.surgery, radiation therapy, systemic treatment, palliative care, rehabilitation). Coordination between the 15 centres included in the pilot project and the coordination centre in Wroclaw within the oncology network created in Lower Silesia Province became an additional aspect.

The centres that form the network in the given province were selected on the basis of quality and organisational criteria, such as the lowest number of procedures performed per year or resources in terms of staff and equipment.

One of the NCN’s primary goals is to introduce a system to monitor the quality of oncological care. For this purpose, registers specific for particular types of cancer were created, and a list of 46 indicators for monitoring the analysed processes was compiled. Twenty-one of these indicators were used to examine the quality of oncological care for lung cancer. Table 1 describes all indicators employed in the Polish National Oncology Network.

**Table 1:** List of indicators employed in the Polish National Oncology Network

Indicator	Description
**F_0**^a^	**Multidisciplinary tumour boards assess the completeness of the diagnostics**
**F_1**	**The percentage of deaths within one year from the diagnosis of a malignant neoplasm, correlated to tumour stage**
**F_2**	**The percentage of deaths within 30 days from the date of surgery, correlated to tumour stage**
**F_3**	**Percentage of deaths within 30 days from the end of chemotherapy, correlated to tumour stage**
**F_4**	**Percentage of deaths within 30 days from the end of palliative radiotherapy, correlated to tumour stage**
**F_5**	**Percentage of patients requiring hospitalisation due to complications after surgical treatment**
**F_6**	**Percentage of patients requiring hospitalisation due to complications after radiotherapy**
**F_7**	**Percentage of patients requiring hospitalisation due to complications after systemic treatment**
**F_8**	**Percentage of patients who received chemotherapy during inpatient hospitalisation**
**F_9**	**Percentage of stage III and IV cancer patients**
**F_10**	**Assessment of the completeness of a pathological exam**
F_11_1	Percentage of patients with genetic and molecular testing for predictive factors (colorectal cancer)
**F_11_2**	**Percentage of patients with genetic and molecular testing for predictive factors (lung cancer)**
F_11_3	Percentage of patients with genetic and molecular testing for predictive factors; immunohistochemistry only (breast cancer)
F_11_4	Percentage of patients with genetic and molecular testing for predictive factors (breast cancer)
F_11_5	Percentage of patients with genetic and molecular testing for predictive factors; FISH only (breast cancer)
F_11_6	Percentage of patients with genetic and molecular testing for predictive factors (ovarian cancer)
F_11_7	Percentage of patients with genetic and molecular testing for predictive factors; immunohistochemistry only (DCIS)
F_11_8	Percentage of patients with genetic and molecular testing for predictive factors (DCIS)
F_12_1	The percentage of surgical procedures performed with minimally invasive surgery (colorectal cancer)
**F_12_2**	**The percentage of surgical procedures performed with minimally invasive surgery (lung cancer)**
F_12_3	The percentage of surgical procedures performed with minimally invasive surgery (ovarian cancer)
F_12_4	The percentage of surgical procedures performed with minimally invasive surgery (prostate cancer)
**F_13**	**Median time elapsed from the date of registration of the patient for a diagnostic (imaging or pathomorphological) exam to the date of obtaining the result of this exam**
**F_14**	**Percentage of repeated diagnostic tests over a 6-week period (computed tomography, endoscopy, biopsy, pathomorphological assessment, molecular assessment), shown for each participating centre by tumour type and test type**
**F_15**	**Percentage of repeated surgical treatments fordiagnoses other thanbreast cancer**
F_16	Percentage of patients with rectal cancer who received preoperative radiotherapy
F_17	Proportion of postoperative histopathology assessment in patients with colorectal cancer with at least 12 lymph nodes assessed
F_18	The rate of anastomotic leakage in colon and rectal cancer
F_19	Assessment of the number of lymph nodes removed during prostatectomy
F_20	The percentage of pelvic lymphadenectomy performedaccording to anatomical ranges
F_21	Amounts of positive postoperative margins after prostatectomy
**F_22**	**Percentage of patients with suspected lung cancer consulted by a pulmonologist within 14 working days from the date of registering the referral with the service provider**
**F_23**	**The proportion of patients with mediastinal lymphadenopathy greater than 10 mm who underwent EBUS-TBNA**
**F_24**	**The proportion of patients with suspected lung cancer and pleural effusion diagnosed with fluid aetiology**
**F_25**	**The proportion of patients with stage III non-small-cell lung cancerwho received concurrent chemoradiotherapy**
F_26	The proportion of ovarian cancer patients treated with primary optimal or suboptimal cytoreduction (no residual mass or ≤ 1 cm)
F_27	The proportion of patients with ovarian cancer who received neoadjuvant chemotherapy
F_28	Percentage of patients with ovarian cancer who underwent exploratory laparotomy
F_29	The proportion of patients with non-infiltrating breast neoplasms not larger than 2 cm in diameter (excluding patients with BRCA1 and BRCA2 mutations) undergoing breast-conserving therapy
F_30	The proportion of patients with infiltrative breast neoplasm not exceeding 3 cm in diameter (total size, including the DCIS component; after excluding patients with BRCA1 and BRCA2 mutations) undergoing breast-conserving treatment
**F_31**	**Percentage of diagnostic tests requiring redescription orreverification of the material over a 6-week period (computedtomography, pathomorphological assessment, molecular assessment),shown for each participating centre by tumour type and test type**
F_32	Percentage of DCIS breast patients with no axillary lymphadenectomy
F_33	The proportion of patients with invasive breast neoplasm withoutlymph node metastases (pN0) without axillary lymphadenectomy
F_34	Percentage of patients with ER-positive and PR-positive infiltrating breast cancer who received hormone therapy
F_35	The percentage of patients with inflammatory or locally advanced, unresectable breast cancer

^a^bold in the text measures used for evaluating lung cancer patients

To ensure a uniform standard of calculations for all indicators, indicator assessment sheets were proposed; these sheets contain data concerning the process to which a given indicator applies, instructions for calculating the indicator, and sources of data and target value (PROQUAL Management Institute). Table 2 provides an example of an indicator assessment sheet.

**Table 2:** Indicator assessment sheet (F_6)

Measure	Percentage of patients requiring re-hospitalization due to complications after radiotherapy											
Characteristics of the measure/indicator	Cancer type (- not applicable, x – applies to):											
	Colorectal cancer (C18, C19, C20)	Lung cancer (C34)		Breast cancer (C50)		Ovarian cancer (C56)		Prostate cancer (C61)		Secondary lung cancer (C78.0)		Preinvasive Brest cancer DCIS (D05)
	X	X		X		X		X		X		X
	Belonging to a type:											
	Describing the state of health (effects of action, depending on the organization)				Statistics (independent of the organization)				Internal process measures (quality)			
	X											
	Link to processes (- none, • weak, •• strong):											
	Diagnosis		Determinig clinical stege				Selection of therapy				Treatment	
	-		-				•				••	
	Measure/Indicator is:											
	Minimant						Maximant					
	X											
	expected value (ideal) of the measure/indicator:											
	0%											
Calculation formula	${\mathrm{M}}_{6}=$ $\frac{\mathrm{number of patients with complications after radiotherapy}}{\mathrm{number of all patients undergoing radiotherapy}}$											
Stratification	1. Clinical stages 2. Cancer type [C18-C20, C34, C50, C56, C61, C78.0 i D05], for each type of cancer—except D05 i C78.0—4 calculations (I, II, III, IV clinical stage) for D05—0 clinical stage (1 calculation) for C78.0—IV clinical stage (1 calculation) In Total: 22 calculations											
Necessary data to calculate the measure	*Source of data*											
Personal Identity Numbers or unique identifiers of patients included in the pilot program	report xml NFZ ^a^/ Hospital Informatic System											
Diagnosis according to ICD-10	report xml NFZ ^a^/ Hospital Informatic System: Colorectal cancer (C18, C19, C20) Lung cancer (C34) Breast cancer (C50) Ovarian cancer (C56) Prostate cancer (C61) Secondary lung cancer (C78.0) Preinvasive brest cancer-DCIS (D05)											
Clinical stage	Hospital Informatic system – set for the day of the Tumor Board											
Last date of radiotherapy course	report xml NFZ ^a^/ Hospital Informatic System											
Information on re-hospitalization and its causes	report xml NFZ ^a^/ Hospital Informatic System Reported treatment complications from radiotherapy or surgery or other reporting. Within 30 days from the last date of radiotherapy Excluding patients whose rehospitalization is associated with undergoing chemotherapy / or hospitalized patients due to injuries, and patients undergoing planned surgery											

^a^NFZ Narodowy Fundusz Zdrowia (Polish)National Health Fund

The Regional Coordination Centre is the unit responsible for calculating the indicators on the basis of clinical (qualitative) and settlement (quantitative) data. These data come from the centres participating in the pilot project in the given province and from the National Health Fund and are stored in a central data warehouse. A uniform electronic format for inputting the data makes it possible to quickly filter the data to calculate specific indicators.

The results are published quarterly. Graphical presentations of the calculated indicators are cumulative quarter-to-quarter and can be accessed through a web application (Oncoindiv3.0).

In the presented results, we decided to focus on three indicators that achieved the expected values and two indicators with results significantly below standards.

## Results

Over the course of the pilot project, in Lower Silesia Province, 3,638 patients—2,161 (59%) men and 1,477 (41%) women—with a diagnosis of lung cancer were included between 2019 and 2021. Patients over 60 were the most numerous group among both men and women, with 1,891 (87%) and 1,267 (85%), respectively included. Of the total number of patients, 420 (12%) were reported by the Regional Coordination Centre (the Wroclaw Comprehensive Cancer Centre), 2,649 (73%) by the Lower Silesia Centre for Pulmonary Diseases in Wroclaw, and 569 (15%) by other centres in Lower Silesia Province.

Expected values were obtained for three indicators. For ‘Percentage of patients who required hospitalisation due to complications following radiation therapy for cancer’, a result of 0% was obtained. For ‘Percentage of diagnostic procedures repeated within a 6-week period (computed tomography, endoscopy, biopsy, pathological analysis, molecular analysis)’, the desired value of 0% was also obtained for the assessed diagnostic procedures, except for pathological analysis, for which a value of 0.05% was derived. The last indicator for which the outcome was consistent with the expected value specified in the indicator assessment sheet was ‘Percentage of diagnostic procedures thatrequired reimpression or reassessment of material within a 6-week period’, with a value of 0% obtained for computed tomography, pathological analysis, and molecular analysis.

For the two indicators, the obtained values were significantly different from the expected values of 100%. For ‘Percentage of patients with suspected lung cancer and pleural effusion in whom aetiology of the effusion was identified’, this aetiology was identified in only 8% of patients. For ‘Percentage of patients with stage 3 non-small-cell lung cancer who received concurrent chemoradiotherapy’, the final outcome was 6%.

A list of all indicators discussed in this analysis, together with the obtained results, is shown in Table 3.

**Table 3:** A list of lung cancer indicators with the obtained results in years 2019–2021

ID	Measure	*N*	Expected value	Obtained values *(year)*		
				**2019**	**2020**	**2021**
F_0^a^	Multidisciplinary tumourboards assess the completeness of the diagnostics^a^	10,793	0%	8%	5%	5%
F_1	The percentage of deaths within one year from the diagnosis of a malignant neoplasm, correlated to tumour stage	505 330 1310 1273	0%	I 1% II 2% III 11% IV 29%	I 4% II 15% III 30% IV 51%	I 4% II 14% III 37% IV 59%
F_2	The percentage of deaths within 30 days from the date of surgery, correlated to tumour stage	362 201 545 492	0%	I 0% II 0% III 0% IV 0%	I 0% II 0% III 1% IV 5%	I 0,3% II 1% III 2% IV 6%
F_3	Percentage of deaths within 30 days from the end of chemotherapy, correlated to tumour stage	71 139 740 665	0%	I 0% II 0% III 6% IV 14%	I 4% II 4% III 11% IV 20%	I 3% II 4% III 12% IV 21%
F_4	Percentage of deaths within 30 days from the end of palliative radiotherapy, correlated to tumour stage	7 17 108 198	0%	I - II- III 0% IV 25%	I - II 0% III 15% IV 21%	I 28%^b^ II 0% III 13% IV 22%
F_5	Percentage of patients requiring hospitalisation due to complications after surgical treatment	2205	0%	1%	1%	5%
F_6	Percentage of patients requiring hospitalisation due to complications after radiotherapy	50 56 316 265	0%	I 0% II 0% III 0% IV 0%	I 0% II 0% III 0% IV 0%	I 0% II 0% III 0% IV 0%
F_7	Percentage of patients requiring hospitalisation due to complications after systemic treatment (after 30, 60, 90 days)	1748 1748 1748	0%	30 d5% 60 d5% 90 d5%	30 d4% 60 d4% 90 d4%	30 d2% 60 d2% 90 d2%
F_8	Percentage of patients who received chemotherapy during inpatient hospitalisation (according to the WHO ECOG)	8818^c^ 156	20%^d^	WHO 0–2 84% WHO 3–4 9%	WHO 0–2 74% WHO 3–4 85%	WHO 0–2 54% WHO 3–4 79%
F_9	Percentage of stage III and IV cancer patients	2417 2417	0%	III 35% IV 32%	III 37% IV 35%	III 37% IV 36%
F_10	Assessment of the completeness of a pathological exam	2167 1857	0% 0%	initial diagnostics0% in-depth 0% diagnostics	initial diagnostics0% in-depth 0,5% diagnostics	initial diagnostics0% in-depth 0,3% diagnostics
F_11_2	Percentage of patients with genetic and molecular testing for predictive factors (lung cancer)^e^	225 211	100% 100%	III 33% IV 77%	III 40% IV 59%	III 35% IV 59%
F_12_2	The percentage of surgical procedures performed with minimally invasive surgery (lung cancer)	724	100%	44%	49%	58%
F_13	Median time elapsed from the date of registration of the patient for a diagnostic (imaging or pathomorphological) exam to the date of obtaining the result of this exam^a^	N/A	0 days (d)	PET-CT 7 d H/P 7 d	PET-CT 12 d H/P 18 d	PET-CT 24 d H/P 14 d
F_14	Percentage of repeated diagnostic tests over a 6-week period (computed tomography, endoscopy, biopsy, pathomorphological assessment, molecular assessment), shown for each participating centre by tumour type and test type	3103 1423 194 1092 3822	0%	CT 0% Biopsy 0% MolecularN/A Endoscopy0% H/P 0%	CT 0% Biopsy 0% Molecular0% Endoscopy0% H/P 0%	CT 0% Biopsy 0% Molecular0% Endoscopy 0% H/P 0,05%
F_15	Percentage of repeated surgical treatments for diagnoses other than breast cancer	2205	0%	2%	2%	6%
F_22†	Percentage of patients with suspected lung cancer consulted by a pulmonologist within 14 working days from the date of registering the referral with the service provider	62	100%	N/A	87%	92%
F_23	The proportion of patients with mediastinal lymphadenopathy greaterthan 10 mm who underwent EBUS-TBNA	217	100%	44%	60%	67%
F_24†	The proportion of patients with suspected lung cancer and pleural effusion diagnosed with fluid aetiology	25	100%	N/A	4%	8%
F_25‡	The proportion of patients with stage III non-small-cell lung cancer who received concurrent chemoradiotherapy	516	100%	3%	1%	6%
F_31	Percentage of diagnostic tests requiring redescription or reverification of the material over a 6-week period (computed tomography, pathomorphological assessment, molecular assessment), shown for each participating centre by tumour type and test type	3103 194 3822	0%	CT 0% Molecular0% H/P 0%	CT 0% Molecular0% H/P 0%	CT 0% Molecular 0% H/P 0%

*N* Number of patients included

H/P Histopathology

CT Computed tomography

^a^Summary data for all diagnoses (lung, breast, prostate, colorectal, and ovarian cancer)

^b^2 patients met the criteria for the measure among 7 patients enrolled

^c^Number of chemotherapy procedures

^d^20% or less

^e^III and IV clinical stages

^†^Indicator for elimination from the NCN

^‡^Indicator requiring modification

## Discussion

One of the network’s main goals is to introduce indicators specific to particular disease entities for use in monitoring the quality of services provided within oncological care. The proposed system is rooted in a mechanism that enables constant improvement under a plan-do-check-act (PDCA) cycle.

The data suggest a correlation between the quality of the medical process, its organisation, and the therapeutic effect [7–10]. Moreover, an idea of quality indicators in oncology is introduced in different areas of cancer care [11–13]. Until now, Poland has had no unified system for managing a large database containing data on cancer patients, which would make it possible to use such data to calculate indicators related to outcomes and internal processes, i.e. the quality of individual hospitals that provide oncological care. The pilot project for the network is meant to test the functioning of specially designed data warehouses, the quality of data stored in these warehouses, the process of collecting the data, and the flow of data between individual components in the network.

When the NCN was being designed, emphasis was placed on creating a unified data structure for all centres participating in the pilot project and on automating processes (e.g.the calculation of individual indicators) to the greatest extent possible. This was done to facilitate and accelerate data processing. In addition, these assumptions meant that the obtained results could be expected to be reliable and transparent and to enable easy comparison between centres or provinces.

The purpose of monitoring oncological care processes is to identify areas that require an increase in quality level or that should be maintained at a specified satisfactory level. Our analysis showed that the results obtained through the NCN indeed identified such areas. Of the 21 indicators, two were at an unsatisfactory level compared to values specified as target values. For ‘Percentage of patients with stage 3 non-small-cell lung cancer who received concurrent chemoradiotherapy’, the obtained result was only 6%. Similar analyses conducted in other European countries revealed a higher percentage of administration of this treatment regimen [14, 15]. Such low results in our province may be related to the different structures of patients with stage 3 cancer included in our analysis. It is possible that the vast majority of patients were stage 3B and 3C patients, who are more commonly qualified for sequential chemoradiotherapy. However, the definition of the indicator failed to differentiate between stages 3A, 3B, and 3C. Another reason for the unsatisfactory outcome may have been organisational or logistical obstacles. Even in Wroclaw, the capital of the province, hospitals that provide systemic treatment for lung cancer patients and the facilities that provide radiation therapy are located in different parts of the city, which could have affected the choice of treatment regimen and led to a preference for sequential therapy. It is also possible that there was a methodological error that affected the reliability of the findings. Further studies are needed to either confirm the above reasoning or to identify other factors that influence the result obtained for the indicator in question. Such internal analysis is currently being conducted by the Regional Coordination Centre.

Increasing the number of patients qualified for concurrent treatment seems vital given the current indications for immunotherapy, which in this instance led to an increase in the overall survival rate in patients who received concurrent chemoradiotherapy [16, 17].

The second outcome, which fell significantly below the set standard, was the very low percentage of lung cancer patients with a diagnosis of pleural effusion at only 8%. Of the 25 patients included in the assessment through this indicator (‘Percentage of patients with suspected lung cancer and pleural effusion in whom aetiology of the effusion was identified’), only 2 received such a diagnosis. However, this indicator received negative opinions from experts who approved it in Lower Silesia Province and will likely not be included in the NCN after the pilot project ends.

One argument supporting the exclusion of this indicator is its doubtful function in the clinical realm. Pleural effusion is typically seen in palliative care patients, for whom delaying symptomatic treatment due to commencement of a pleural effusion diagnosis seems unacceptable.

Furthermore, the number of included patients for this indicator was very low at only 25. The same was true for ‘Percentage of patients with suspected lung cancer seen by a pulmonary specialist within 14 working days of registering a referral with the health care provider’ at only 62 patients. This may have been caused by incorrect design of the indicators and the lack of information concerning the described event (such as being seen by a pulmonary specialist) in the patient’s medical history, which makes it impossible to correctly assign the patient within the indicator in question. This indicator may also be rejected following the conclusion of the pilot project according to the reasons stated above.

Analysis of the results obtained through the NCN for our province also identified indicators that met their target values or were very close. Two of these described the patients’ condition. The first measured the share of patients who required hospitalisation due to complications following the conclusion of radiation therapy for cancer and had a value of 0% at the end of the assessed period. The second evaluated the number of patients who required hospitalisation due to complications following systemic treatment. In this case, the data concerned the periods of 30, 60, and 90 days after the assessed therapy ended. A result of 2% was obtained for each period. When compared to similar analyses, the obtained outcomes may be regarded as indicating that the frequency of complications requiring hospitalisation following non-surgical treatment in our region is optimal [18, 19].

We believe, however, that the introduction of an additional indicator—which would make it possible to assess the number of hospitalisations required during radiation therapy administered primarily on an outpatient basis—should be considered. Other new indicators could also be included in the NCN, e.g. indicators evaluating aspects related to immunotherapy or stereotactic radiation therapy for lung cancer.

Another group of indicators that met the levels expected under the assumptions of the NCN involves diagnostic procedures. These indicators describe internal processes and the quality of specific procedures. Therefore, the percentage of imaging, endoscopic, and molecular procedures that required reimpression or reassessment or were repeated within a 6 week-period was 0%. This outcome reflects the high quality of initial and follow-up diagnostic procedures for lung cancer in centres that form a part of the NCN in the analysed region of Poland.

It is worth referring to an analysis of tests performed for genetic and molecular predictors in patients at clinical stages 3 and 4 and the epidemiological structure of this group of patients. Compared to the findings presented in that analysis, which cover the NCN’s period of functioning between 2019 and 2020 in Lower Silesia Province, the current study showed no significant differences in the scope of the analysed aspects [20]. Only the percentage of stage 3 patients who underwent an assessment of predictors decreased, from 40% at the end of 2020 to 35% at the end of 2021.

A significant disproportion between the number of lung cancer patients reported by individual centres is noticeable. It stems from the specific nature of the organisation of health care related to pulmonary diseases in Lower Silesia Province. As many as 73% of patients included in the pilot project for the NCN were reported by a single centre, the Lower Silesia Centre for Pulmonary Diseases in Wroclaw. This unit has been the primary centre for the treatment of pulmonary diseases in our region for many years, which explains the high number of patients reported by it among those included in the pilot project.

From the perspective of the idea of the project, a sufficiently high level of inclusion of patients in the network is essential. Leaving patients out of the network leads to results that may provide an inadequate picture of the current situation as assessed by a particular indicator. Likewise, consistent reporting of patients by the same centre or within the same province is key with respect to the reliability of the outcomes. In our analysis, we noticed differences in the number of reported clinical stage 3 patients for two indicators: the statistical indicator that evaluated the percentage of stage 3 patients encompassed 890 patients, while the indicator that assessed the share of patients in the same stage who received concurrent chemoradiotherapy had only 516 patients. The cause of this difference can be traced to different definitions of indicators and criteria for inclusion. It also seems likely that, simultaneously, there may have been errors in reporting caused by imprecise information contained, e.g. in medical histories of patients included in the pilot project or by patients ‘escaping’ the NCN due to errors in the data management process, which was not automated.

It is clear that working with such a large database concerning cancer patients requires many years of observation. This condition allows us to assume that it will be possible to correlate results obtained through the NCN during its operation for individual types of malignancies with direct treatment effects, expressed through a local control group or the overall survival rate. Furthermore, identifying such correlations will make it possible in the future to modify specific steps of oncological care and thus create conditions to improve the effectiveness of cancer treatment in a given area. Adopting the NCN concept into the Polish oncological care system would create an opportunity.

At the moment, it is difficult to compare the outcomes obtained and presented in this paper with the period before the pilot project was implemented for the NCN, as Poland had no similar system for quality assessment in oncology in place.

This paper presents findings on a selective basis, concentrating on the analysis of indicators related to lung cancer diagnosis and treatment. Other components of the NCN—such as the creation of the position of Coordinator for Oncological Care; measurement of the level of satisfaction with health care, assessed by the patient using a dedicated survey; or standardisation of reports from pathological and radiological exams—require further, separate analyses.

The limitation of the presented work is the pilot nature of the project. Errors in data reporting, incomplete data, and elements of the NCN, such as the aforementioned measures that do not fulfill the intended role, are some of the limitations of the pilot.

We believe that the ongoing pilot project for the NCN, understood as a platform for testing the NCN’s components, will make it possible to identify significant limitations; once these are eliminated, we can take advantage of the potential stemming from coordinated and high-quality oncological care.

## Conclusions

The pilot project for the NCN is an ambitious and innovative for Poland as a whole, aimed at assessing the functioning of a network of oncological centres and managing data used to calculate quality indicators for oncological care. The analysis shows the results of the operations of the NCN in Lower Silesia Province for lung cancer. Further examination of the NCN spanning its entire 3-year period—including a focus on the shortcomings and irregularities in its functioning with a view to eliminating them in the future—will prove invaluable.

The NCN in Poland offers opportunities to improve the quality of cancer care, including diagnostics and treatment, and to select centers that meet the established standards. In our opinion, this may translate into an improvement in the results of oncological treatment in the future.

## Supplementary Information

**Additional file 1.**

## Data Availability

The data underlying this article will be shared on reasonable request to the corresponding author.

## Abbreviations

NCN: National Cancer Network
KSO: Polish National Oncology Network (Krajowa Sieć Onkologiczna, polish)
PDCA: Plan-Do-Check-Act
